# Alternative splicing in addiction

**DOI:** 10.1016/j.gde.2025.102340

**Published:** 2025-03-19

**Authors:** Akanksha Bhatnagar, Elizabeth A Heller

**Affiliations:** 1 Department of Systems Pharmacology and Translational Therapeutics, University of Pennsylvania, Philadelphia, PA 19104, USA; 2 Institute for Translational Medicine and Therapeutics, University of Pennsylvania, Philadelphia, PA 19104, USA; 3 Penn Epigenetics Institute, Perelman School of Medicine, University of Pennsylvania, Philadelphia, PA 19104, USA

## Abstract

Addiction is a chronic and relapsing medical condition characterized by the compulsive use of drugs or alcohol despite harmful consequences. While transcriptional regulation has long been recognized for its role in addiction, recent genome-wide analyses have uncovered widespread alternative splicing changes that shift protein isoform diversity in multiple brain reward regions central to addiction. In this review, we discuss emerging research and evidence that alternative splicing is dysregulated in cocaine, alcohol, and opioid use disorders.

## Introduction

Addiction is a chronic and relapsing medical condition characterized by the compulsive use of drugs (substance use disorder [SUD]) or alcohol (alcohol use disorder [AUD]) despite harmful consequences [1]. These disorders are marked by an inability to control the urge to consume substances and continued intake despite repeated attempts to quit [2]. Addiction imposes a serious public health concern in the United States, with 17.1% of the population aged 12 years and older meeting the criteria for SUD and 10.2% for AUD in 2023 [3]. The prior literature establishes the functional relevance of neuronal alternative splicing in the contexts of neuronal activation [4,5], human development [6], autism spectrum disorder [7], paraneoplastic disease [8], and neurodegenerative disorders [9]. However, there is still a gap in understanding the regulation of alternative splicing in psychiatric disorders, including addiction.

**Figure 1:**
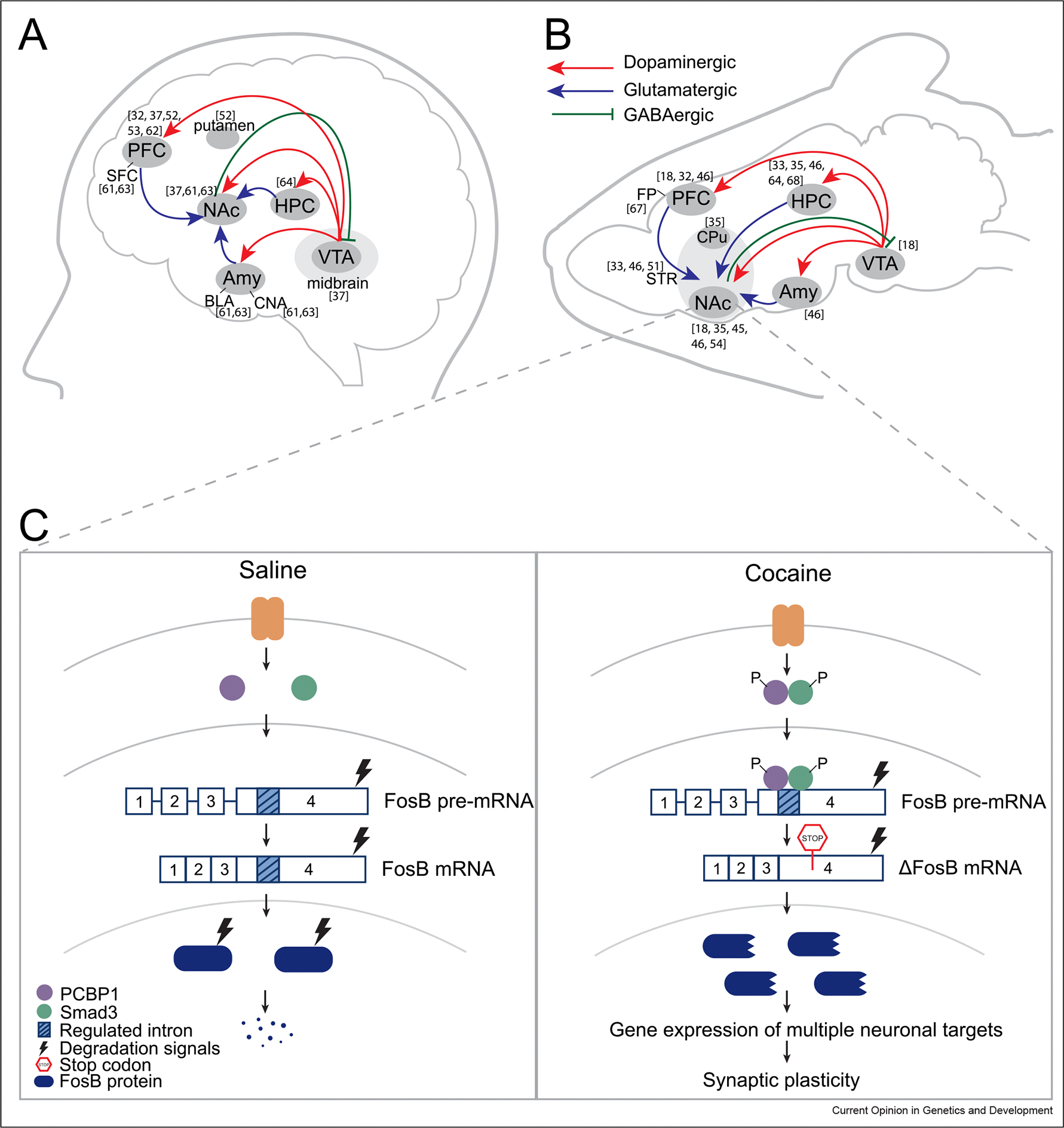
**(a, b)** Reward circuit in the human **(a)** and the rodent **(b)** brain. NAc receives dopaminergic neurons from the VTA and glutamatergic neurons from PFC, HPC, and Amy. NAc projects inhibitory GABAergic neurons into the VTA. Abbreviations: FP: frontal pole. **(c)** Alternative splicing of *fosB* gene into ΔFosb mRNA is a common splicing event in multiple substances of abuse. PCBP1 splicing regulator along with Smad3 binds to Fosb pre-mRNA favoring exclusion of the regulated intron 4. The resulting ΔFosb mRNA contains an in-frame stop codon preceding the protein degradation signals and is therefore translated into a truncated, highly stable ΔFosb protein form that persists for weeks after drug exposure. ΔFosb then regulates gene expression of several downstream targets associated with synaptic plasticity to strengthen neuronal connections mediating reward stimuli.

Despite distinct pharmacological targets, all drugs of abuse drive reinforcing behavior through activation of the mesolimbic reward circuit in humans, rodents, and model organisms (Figure 1a,b) [2,10]. Dopamine lies at the center of this reward circuit. Dopaminergic neurons originate in the ventral tegmental area (VTA) in the midbrain and project to forebrain targets, including nucleus accumbens (NAc), prefrontal cortex (PFC), hippocampus (HPC), and amygdala (Amy) [2]. Stimulants, such as cocaine and amphetamine, directly increase dopaminergic transmission by blocking dopamine reuptake transporter in the NAc [2]. In contrast, opioids indirectly increase dopamine release by binding to opioid receptors on inhibitory interneurons in the VTA, which in turn disinhibit dopaminergic neurons projecting to the NAc [11]. Similar to opioids, one of the key mechanisms for alcohol is enhancing gamma-aminobutyric acid (GABA_A)_ receptor function that also disinhibits dopaminergic transmission to NAc [11]. Opioids can also directly act on NAc neurons by stimulating mu opioid receptors (MORs), further boosting dopamine transmission in NAc [11]. Thus, irrespective of the drug-specific cascade, all drugs of abuse hijack the reward circuit to increase dopamine release in the NAc that underlies the reinforcing effects of drugs [11,12].

Drug addiction triggers long-term changes in the brain at the molecular level, affecting the transcriptome [10,13]. These changes occur both at the transcriptional level affecting gene expression and through post-transcriptional regulation, such as alternative splicing [13]. Alternative splicing is a fundamental process that allows a single gene to generate multiple mRNA splice variants by rearranging exons and introns, leading to protein isoforms with unique functions [14,15]. Alternative splicing affects more than 90% of multi-intronic human genes and is particularly abundant and conserved in the brain [9,16]. Neuronal alternative splicing provides an extended proteome essential for fine-tuning brain function in order to respond and adapt to different stimuli [17]. Interestingly, neural genes undergoing splicing changes rarely overlap with genes with altered gene expression [7,18–21], suggesting neurons respond to stimuli via parallel signaling pathways that alter either transcription or alternative splicing to shift the protein amount or protein isoform diversity, respectively.

Recent advances in next-generation sequencing have revolutionized our ability to analyze gene expression and alternative splicing at an unprecedented depth and resolution [22]. Short-read RNA-sequencing, in combination with splicing analysis tools such as rMATS [23], LeafCutter [24], and MAJIQ [25], has been instrumental in identifying differential splicing events across various conditions and cell types. Now, long-read sequencing marks a significant leap forward in capturing the entire splicing events within full-length transcripts by obviating the need for cDNA fragmentation [26,27]. In this review, we discuss emerging research and evidence that alternative splicing is dysregulated in cocaine, alcohol, and opioid use disorders (OUDs) [28].

### Alternative splicing in opioid use disorder

Exogenous opioids, such as morphine, heroin, and fentanyl, activate the MOR encoded by Opioid Receptor Mu 1 (*OPRM1*). *OPRM1* undergoes extensive alternative splicing to form 21 distinct MOR isoforms with clinically relevant differences in opioid responsivity and analgesia [29,30]. In addition to direct receptor binding, opioid alters OPRM1 pre-mRNA splicing to produce distinct MOR isoforms with varying binding affinities, receptor activation, and downstream signaling, ultimately influencing opioid analgesic efficacy and side effects [31]. OPRM1 undergoes alternative splicing in the medial PFC of heroin self-administering male rats and male human heroin abusers, showing conservation of morphine-mediated OPRM1 splicing [32]. Acute subcutaneous administration of morphine alters splicing of Oprm1 from the canonical MOR-1 to MOR-1X variant in the HPC and striatum (STR) of mixed-sex rats that distinctly activates mitogen-activated protein kinase (MAPK) signaling, likely impacting morphine-mediated analgesia [33]. In contrast, a morphine-induced conditioned place preference (CPP) paradigm is used to study chronic dosing with repeated injections that is persistent over time and can be reinstated by morphine after extinction. A morphine CPP paradigm finds transient alternative isoform expression of brain-derived neurotrophic factor (BDNF) in the HPC, NAc, and caudate putamen (CPu) of male mice. Specifically, BDNF splice variants II, IV, and VI increase during the acquisition of morphine preference suggesting a role in learning and memory but are short lived and return toward baseline during drug extinction and reinstatement [34]. Since BDNF modulates GABAergic activity–mediated neurotransmission in the reward circuit [35], these BDNF splice variants may contribute to neuronal signaling and growth. Although there is no known mechanistic link between Oprm1 and BDNF splicing, both these events contribute to opioid dependence.

Although the effect of opioids on splicing of OPRM1 and other single targets are well characterized, genome-wide analyses of opioid-induced alternative splicing have only recently emerged. Huggett et al. [36] compared alternative splicing in dorsolateral PFC (50% female), NAc (50% female), and midbrain (100% female) in the postmortem brain of 90 OUD patients and their matched controls. A total of 1788 differential splicing events, about half of which were exon skipping, in 788 differentially spliced genes (DSGs) were associated with chronic opioid use across brain regions [36]. Notably, although chronic opioid use was also associated with 922 differentially expressed genes (DEGs), only 3% of DEGs were also differentially spliced [36]. Differential splicing was largely brain region specific, but five DSGs were present across all three brain regions: SNHG14, HERC1, HILPDA, METTL2B, and BIN1. Across all samples and brain regions, BIN1 (Bridging integrator 1 or Amphiphysin 2) shows consistent opioid-associated splicing in the clathrin and AP-2-binding (CLAP) domain, which facilitates clathrin-mediated endocytosis [36]. This splicing event in the CLAP domain may alter MOR receptor endocytosis and desensitization resulting in reduced drug responsiveness and promoting opioid tolerance. Additionally, the CLAP domain is included only in the neuronal isoforms of BIN1, and its skipping is associated with reduced Aβ endocytosis and clearance-mediated neurotoxicity in Alzheimer’s disease [37,38], warranting further investigation into BIN1 isoform switching in OUD. Finally, spliceosome genes, such as U1 and U2 small nuclear RNAs, were upregulated and perturbations in the spliceosome pathways were enriched in OUD brains [36], suggesting spliceosomal dysregulation could underlie splicing changes in opioid addiction. In contrast to short-lived splicing changes detected via polymerase chain reaction (PCR) after chronic morphine administration in mice, postmortem human brains from OUD patients with a history of opioid misuse reveal widespread splicing alterations even after death, suggesting prolonged opioid exposure and RNA-sequencing techniques might improve our ability to detect splicing changes [36].

### Alternative splicing in cocaine treatment

Cocaine is a psychostimulant that blocks dopamine reuptake from the synaptic cleft, resulting in increased dopamine postsynaptic signaling [39]. Chronic cocaine exposure induces persistent changes in synaptic structure and function [40] through global changes in gene expression [41,42] and the epigenome [43,44]. The effect of cocaine treatment on alternative splicing is evident by studies focusing on single gene targets after chronic or acute drug exposure. Over 30 years ago, Nestler et al. found that cocaine exposure in humans and animals leads to the alternative splicing of Fosb transcription factor into ΔFosb in neurons in NAc [45]. ΔFosb initiates and sustains expression of multiple downstream targets, such as Cdk5, NFκB GluA2, Gria2, and CAMKII, to promote dendritic spine formation and regulate synaptic plasticity [46]. Accordingly, ΔFosb accumulation has been implicated in as critical neurobiological functions, including learning and memory and aggression phenotype [46,47]. Today, ΔFosb accumulation is a common addiction link observed for cocaine, morphine, amphetamine, alcohol, nicotine, and phencyclidine, making it a critical molecular switch in SUD [47,48]. Despite the long-established role of ΔFosb in addiction, the mechanism of splicing was only recently elucidated. Krapacher et al. [49] find that acute cocaine administration activates PCBP1, an RNA splicing regulator, that favors the exclusion of intron 4 in Fosb pre-mRNA resulting in a stop codon preceding the protein degradation signals in the ΔFosb mRNA. Upon translation, the truncated ΔFosb splice isoform is highly stable [49] and activates gene expression that induces synaptic changes in NAc promoting drug-seeking behavior (Figure 1c) [47].

Beyond ΔFosb, only scant data are available on specific gene targets spliced in cocaine use disorder. Unlike chronic morphine administration, acute cocaine administration but not repeated drug exposure induces BDNF IV splice variant in the rat striatum transiently for promoting neuron growth in the NAc [50]. Additionally, in human cocaine abusers, alternative splicing of dopamine receptor D2 reducing formation of D2 short isoform [51] and reduced expression of a truncated isoform of serotonin 2A receptor (HTR2A) [52] likely affecting memory processing and cocaine dependence. Moreover, repeated investigator-administered cocaine injections in male mice induce splicing of the transcription factor E2F3 to produce E2F3a isoform that regulates both transcription and splicing of key cocaine response targets, including Ptbp1 splicing regulator, Fgfr1 growth factor, and Tle2 transcriptional corepressor [53]. Remarkably, E2F3a overexpression is sufficient to recapitulate gene expression and splicing changes in NAc caused by cocaine, establishing E2F3a as a novel upstream regulator of cocaine action in NAc [53].

With recent advances in sequencing technology, genome-wide alternative splicing changes have now been associated with cocaine treatment across brain reward regions [18,44]. RNA-sequencing of NAc in male mice after repeated investigator-administered cocaine treatment finds far greater changes in alternative promoter usage and alternative splicing (2998 DSGs) than differential expression (92 DEGs) [44]. Using a cocaine self-administration paradigm on mixed-sex mice, we also find widespread differential alternative splicing of 339, 369, and 799 DSGs in the NAc, PFC, and VTA, respectively [18]. Since cocaine self-administration in mice involves volitional drug intake, cognitive learning to obtain the drug and faster absorption intravenously, these splicing changes [18] are expected to better mimic drug-seeking behavior in humans than repeated investigator-administered treatment [44,53]. Only four cocaine-driven DSGs are common to all three brain regions, and there is negligible overlap between any two brain regions, suggesting a high degree of regional specificity [18]. Notably, cocaine-induced alternative splicing of the serine- and arginine-rich splice factor Srsf11 and Srsf11 motifs is highly enriched at exon junctions across DSGs, making Srsf11 a putative splicing factor regulating cocaine-driven alternative splicing [18]. Of note, Srsf11 regulates splicing of Cacna1b gene into calcium channel CaV2.2 that regulates neurotransmitter release and has been implicated in cocaine reward behavior and nociception [18].

It is well established that epigenetic changes underlie cocaine-driven differential gene expression. An emerging literature suggests that epigenetic changes may also drive alternative splicing [65–68]. We find that the histone modification, H3K36me3, is enriched at cocaine-driven alternative exons, but not at constitutive exon junctions, implicating a role for chromatin in cocaine-induced alternative splicing [18]. Furthermore, to distinguish a direct role of H3K36me3 in splicing via recruitment of splicing machinery from an indirect role via altered splice factor expression, we apply targeted epigenetic editing to enrich H3K36me3 specifically at *Srsf11* splice junctions [18]. *Srsf11*-targeted H3K36me3 enrichment is sufficient to drive splicing of *Srsf11* and partially recapitulate cocaine-induced DSGs genome wide, as well as to enhance cocaine-reward behavior. Taken together, these data support a direct functional role of H3K36me3 in cocaine-driven alternative splicing [18].

### Alternative splicing in alcohol use disorder

Alcohol exposure produces wide-ranging effects on intracellular signaling and molecular mechanisms resulting in pan-neuronal adaptations that underlie AUD [69]. To advance the knowledge of alcohol-induced global changes in gene expression [70] and characterize alcohol-induced genome-wide changes in alternative splicing, Van Booven et al. [54] performed RNA-Seq on postmortem human AUD brain tissues (77% male). In contrast to just 23 DEGs, profound mis-spliced events were observed in all four brain regions studied (1421 events in superior frontal cortex [SFC], 394 in NAc, 1317 in basolateral amygdala [BLA], and 469 in central nucleus of amygdala [CNA]) [54]. Importantly, the DSGs displayed high-regional specificity with only 14 DSGs overlapping in all four brain regions [54]. These alcohol-induced splicing abnormalities were attributed to the increased expression of the splicing factor HSPA6 and aberrant expression of long noncoding RNAs, but expression of small nuclear RNAs involved in the spliceosome was unaffected [54]. Interestingly, another AUD study revealed exon skipping events in ELOVL7, LINC00665, and NSUN4 that are upstream of HSPA6 splicing factor, to be a risk factor for AUD, supporting a role for HSPA6 in global mis-splicing across the brain [56]. Furthermore, Huggett et al. [55] reanalyzed the Van Booven et al.’s [54] AUD data set to investigate genetic links underlying alternative splicing in AUD. The 713 DSGs identified were enriched for neurotransmission, intracellular signaling, and drug/alcohol metabolism and did not overlap with the 53 DEGs [55]. Additionally, 6463 splicing quantitative trait loci that are specific genetic variants associated with DSGs in AUD were observed across the four brain regions, suggesting genetic contributions of alternative splicing in AUD [55]. In a chronic 15-day ethanol exposure study, upregulation of the PCBP1 splicing factor was associated with enriched binding and increased intron retention of Hapln2 only in the male rat HPC, suggesting sex differences in alcohol-induced alternative splicing [57]. The Hapln2 splicing event is predicted to result in a truncated protein with loss of function for nerve conduction velocity, therefore affecting HPC function in AUD [57]. In contrast, splicing factors SRSF1 and SRSF11 were enriched after chronic ethanol exposure in male monkeys that is relevant to human AUD [71]. Similarly, a study on alcohol-associated liver disease in humans and chronic-binge alcohol treatment in mixed-sex mice reveals SRSF10 splicing factor to be critical for favoring production of lipin 1β isoform that increases liver lipid production and contributes to disease progression [58]. Consistent with the splicing studies in humans, ethanol-induced behavioral sensitization in male mice led to differential exon usage of 1067 exons in 746 genes relevant to mRNA processing, protein stability and translation, and synaptic function in the synaptoneurosome [59]. Although this study found little to no splicing perturbations after acute ethanol exposure in frontal pole (42 exons in 36 genes, synaptoneurosome) [59], another study found 13 770 exons to be differentially expressed after acute ethanol treatment in the HPC of male mice [60].

Similar to a developed adult brain, alcohol exposure has widespread implications on alternative splicing during early nervous system development as well [61–64]. Kawasawa et al. [61] performed RNA-Seq on ethanol-exposed fetal human female cortex to identify genome-wide splicing alterations. A total of 382 (174 novel) alcohol-induced alternative splicing events were discovered with intron retention as the most common splicing event [61]. To further uncover the effect of prenatal ethanol exposure on alternative splicing, Fuentes-Beals et al. [62] used four different ethanol exposures on early development data sets from mice and human to predict alcohol-induced splicing perturbations. Of the eight common genes that were predicted to be alternatively spliced by all splicing analysis tools in human embryonic cortex, four genes (CHD2, HNRNPH1, SF1, WTAP) are relevant to splicing regulation, suggesting ethanol exposure affects the control of splicing process [62]. Importantly, pathway-enriched analysis revealed that the genes involved in RNA processing and protein synthesis were frequently alternatively spliced in different data sets [62], supporting ethanol-induced splicing abnormalities during early development. When fetal neurons were exposed to ethanol, expression of the splicing factor SRSF1 was drastically reduced, resulting in mis-splicing of its downstream target, anti-apoptotic myeloid cell leukemia 1 (Mcl-1) [63] that is suggested to reduce neuron viability and mediate alcohol exposure–associated neurotoxicity [72]. Finally, in prenatal ethanol exposure of mixed-sex mice, transcript-wide changes in alternative splicing were observed that have the potential to be utilized as peripheral biomarkers for early prediction of motor learning deficits [64].

## Conclusion and future directions

Alternative splicing has now emerged as a shared molecular brain adaptation in addiction (Table 1) [28]. Genome-wide studies have identified global alternative splicing changes in multiple brain reward regions associated with opioid [36], cocaine [18,44], and alcohol treatment [54,55,59,61,62]. These splicing changes show a high degree of regional specificity, suggesting that different neural circuits and processes may be uniquely affected by substance use. Additionally, the negligible overlap between genes undergoing expression and splicing changes suggests independent signaling pathways regulate transcriptional and post-transcriptional processes. Despite differences in aberrantly spliced genes by different drugs of abuse, two emerging common pathways include (1) neuronal signaling and neurotransmission: MAPK [33], dopamine [51], serotonin [52], AMPA [55], GABA [59], NMDAR [60], and calcium signaling [18,59]; (2) RNA transcription and processing: ΔFosb [45,49], E2f3a [53], Srsf11 [18], Srsf5 [59], SF1, and WTAP [62].

Despite these recent advancements, a comprehensive understanding of causes and consequences of these splicing events remains limited, restricting therapeutic intervention in addiction. Although several splicing factors, such as HSPA6 [54], PCBP1 [49], SRSF1/10/11 [18,71,58], and H3K36me3 [18] epigenetic modification have been implicated, additional studies are needed to validate their functional relevance in addiction and identify upstream regulatory mechanisms governing drug-induced alternative splicing. Current splicing therapeutic tools are limited by the need to deliver DNA expression (trans-splicing and modified small nuclear RNAs) or lack of specificity (small molecules) [73]. Alternatively, splice-switching antisense oligonucleotides (ssASO) are currently being explored for highly specific, low toxicity, and ease of delivery for treatment of neurological diseases [74,75]. An ssASO hybridizes to splice-factor recognition-specific sequences within target pre-RNA to either block or promote splicing. Putative ASO splice-switching targets include OPRM1 [32,33], BDNF [34], and BIN1 [36] for opioid; BDNF [50], HTR2A [52], Srsf11 [18], and E2F3 [53] for cocaine; ELOV7 [56], GRIA2 [55], Hapln2 [57], and GRIN1 [60] for alcohol treatment. The recent discovery that PCBP1 drives splicing of the ‘addition switch’, ΔFosb [49], represents a promising ssASO target in SUD [47,48].

Moreover, these findings underscore the need for in-depth studies analyzing neural alternative splicing in response to other substances of abuse to delineate similarity and differences between their mechanisms. So far, lysergic acid diethylamide has been shown to increase the number of splicing junctions used in rat mPFC [76], while methamphetamine decreases total splicing events in the mice brain [77]. Additionally, cannabinoid exposure mis-splices Npas2 transcription factor and Hdac4 histone deacetylase [78], while nicotine exposure mis-splices NEAT1 lncRNA likely affecting mRNA transport [79]. These PCR studies reveal splicing changes with multiple drugs of abuse but limit our ability to determine splicing mechanisms. Finally, most addiction studies primarily use male subjects or mixed sex, limiting our understanding of sex-specific differences in splicing. Since sex differences in alcohol-induced alternative splicing have been speculated [57], future studies must explore splicing changes in both sexes.

## Figures and Tables

**Table 1:** Drug-induced alterations in RNA alternative splicing.

Drug class	RNA-Seq splicing analysis?^a^	Major splicing alterations		Potential functional implications	Model organism and tissue	Ref
		Gene	Splice variant			

Opioid-morphine	–	OPRM1	↑ MOR-1X	MAPK signaling	Mice: HPC, STR	[33]
Opioidheroin	–	OPRM1	↑ hMOR-1X, hMOR-1H, hMOR-1G2 ↓ hMOR-1A, hMOR-1B2	Biased G protein-coupled receptors(GPCR) agonism between β-arrestin and G-protein; Heroin analgesia	Human: mPFC	[32]
			↑rMOR-1G1 ↓ rMOR-1A, rMOR-1B1		Rat: mPFC	
Opioid-morphine	–	BDNF	↑ BDNF II, IV, VI	Neuronal growth; learning and memory	Mice: HPC, NAc, CPu	[34]
Opioid-fentanyl, heroin, oxycodone	Yes, LeafCutter	BIN1	Alternative splicing of CLAP domain	Protein phosphorylation, early endosome, GTPase activator activity	Human: dlPFC, NAc, midbrain	[36]
Cocaine	Yes, Cuffdiff	Ttc23, Sp100, Sept7	↑ exon inclusion, ↑ exon inclusion, ↓ exon inclusion	Nucleotide and ion binding, protein localization	Mice: NAc	[44]
Cocaine	–	Fosb	↑ ΔFosb lacking intron 4	Transcriptional activation	Mice: NAc, PFC, HPC, dSTR, Amy	[45]
Cocaine	–	BDNF	↑ BDNF4	Activate tyrosine receptor kinase B, promote neuron growth	Rat: STR	[50]
Cocaine	–	D2 receptor	↑ D2 long	Dopamine signaling; memory processing	Human: PFC, putamen	[51]
Cocaine	–	*HTR2A*	↓ truncated exon 2	Serotonin signaling; cocaine reinforcement and sensitization	Human: dlPFC	[52]
Cocaine	Yes, rMATS and MAJIQ	Srsf11, Cacna1b, Shisa	↑ inclusion of bleeding exon	Splice factor; calcium voltage-gated channel, GABA signaling	Mice: NAc, VTA, PFC	[18]
Cocaine	Yes, rMATS	E2F3, Tle2, Ptbp1	↑ E2f3a, ↓ exon 10, ↑ exon 8	Transcriptional activator, splice regulator	Mice: NAc	[53]
Alcohol	Yes, rMATS, LeafCutter	GRIA2	↑ Exon 14 skipping	Alpha-amino-3-hydroxy-5-methyl-4-isoxazolepropionic acid (AMPA) receptor neurotransmission, intracellular signaling, and drug/alcohol metabolism	Human: SFC, NAc, BLA, CNA	[54,55]
Alcohol	Yes, rMATS	ELOVL7	↑ skipped exon in 5’UTR	Innate immune system	Human: dlPFC	[56]
Alcohol	Yes, edgeR exactTest()	Hapln2	↑ A3SS in exon 4	Extracellular matrix (ECM) component; Nerve conduction velocity	Rat and human: HPC	[57]
Alcohol	–	Lipin 1	↑ lipin 1β	Increased lipid production in liver	Human, Mice: liver	[58]
Alcohol	Yes, DEX-Seq	Spag9, Cacnb1,Srsf5	–	synaptic signaling, mRNA processing	Mice: frontal pole	[59]
Alcohol	Yes, DEX-Seq	Grin1	Exons 4, 5, 6, and 8	NMDAR1 synaptic function	Mice: HPC	[60]
Alcohol	Yes, juncBASE	SHANK2, PTPRD	↑ exon skipping, ↑ exon skipping	Cell death, apoptosis, and cell junctions	Human: fetal cortex	[61]
Alcohol	Yes, VAST-TOOLS, rMATS, MAJIQ	SF1, CHD2, WTAP	–	RNA processing, chromatin remodeling	Human and mice: fetal cortex, embryonic cells	[62]
Alcohol	–	Mcl-1	↑ Mcl-1S	Anti-apoptotic; neurotoxicity	Human: fetal neurons	[63]
Alcohol	Yes, rMATS, LeafCutter	Kdm7a, Usp15, Dapp1, Brox		Early brain development and function	Mice: peripheral blood mononuclear cells	[64]

a RNA-Seq splicing analysis revealed transcript-wide DSGs, only major splicing alterations are shown here.

## Data Availability

No data were used for the research described in the article.
